# Dynamic Analysis and Prediction of Food Nitrogen Footprint of Urban and Rural Residents in Shanghai

**DOI:** 10.3390/ijerph17051760

**Published:** 2020-03-08

**Authors:** Yuling Xia, Chengsong Liao, Dianming Wu, Yanzhuo Liu

**Affiliations:** 1 Key Laboratory of Geographic Information Science (Ministry of Education), School of Geographic Sciences, East China Normal University, Shanghai 200241, China; 2 Institute of Eco-Chongming (IEC), East China Normal University, Shanghai 202162, China; 3 Institute of Xilingol Bioengineering Research, Xilingol Vocational College, Xilinhot 026000, China

**Keywords:** food nitrogen footprint, urban residents, disposable income, Engel’s coefficient, sustainable development goals

## Abstract

The food nitrogen (N) footprint reflects the amount of reactive N emission and its impact on the environment as a result of food production and consumption to satisfy the basic food demands of an urban population. The N-Calculator model was used to estimate the food N footprint and its dynamic changes in Shanghai from 2000 to 2017, and the auto regressive integrated moving average (ARIMA) time series model was used to predict the food N footprint in Shanghai from 2018 to 2027. The results show that the food N footprint was higher in urban areas (15.3–18.8 kg N/capita/yr) than rural areas (12.6–17.4 kg N/capita/yr) of Shanghai from 2000 to 2017. The change in the food N footprint was consistent with changes in food consumption in urban and rural areas, and the total food N footprint of urban and rural residents was positively correlated with the per capita disposable income and population whereas it was negatively correlated with the Engel’s Coefficient and price index. It was predicted that the per capita food N footprint will gradually decrease in 2018–2027 in urban areas of Shanghai, but it will generally increase in the rural areas. This study will help to initiate policy interventions for sustainable N management and contribute to the achievement of key sustainable development goals (SDGs).

## 1. Introduction

Along with economic improvement and the rapid development of urbanization in China, the demand for food has also grown rapidly and the corresponding consumption structure has diversified [1,2]. In the process of food production and consumption, a large amount of nitrogen (N) is released into the natural environment, which causes an N imbalance in ecosystems [3]. It has been found that the loss of reactive N generated by food production and consumption has a major influence on the N footprint; this is one of the key environmental issues in China [4]. N is an essential element for biological growth, however, several ecological and environmental problems are caused by excessive N, such as pollution from agricultural non-point sources and the extinction of aquatic life [5,6,7]. Statistical analyses of the food N footprint are helpful for evaluating the impact of human production and lifestyle on the ecological environment, so as to provide theoretical support for reducing nitrogen emission intensity and optimizing the ecological environment in the future. The information also contributes to policy interventions for sustainable N management and to the achievement of key sustainable development goals (SDGs).

The N footprint, which was first proposed at the 5th International Nitrogen Conference in 2010, has been defined as the sum of the reactive N emitted directly or indirectly by products or services during their production, transportation, storage, and consumption [8,9,10]. An N-Calculator model is used to calculate the N footprint at the national, household, and personal scales. The N footprint model was developed by Leach et al. [10], and it provides information on how individual and collective action can result in the loss of reactive N to the environment. The N-Calculator focuses on food and energy consumption and uses average per capita data at for the country scale, while at the personal level, it is based on individual resource consumption [10]. To date, the N footprints for Germany, the Netherlands, the United States, and the United Kingdom have been calculated using the N-Calculator model and these are 24, 23, 39, and 27 kg N/capita/yr, respectively [10,11]. It was also found that more than 70% of the N footprint is derived from food-related activities [10,11]. In addition, the estimated food N footprint of urban residents in Beijing was 14.7–22.6 kg N/capita/yr from 1980 to 2012, while that of rural residents was 10.8–15.3 kg N/capita/yr according to the N-Calculator model [12]. Moreover, the food N footprint in Guangzhou from 1980 to 2009 was 25.3–35.0 kg N/capita/yr [13]. However, very few studies have focused on the food N footprint of residents in Shanghai, which is a rapidly developing city that is representative of the Chinese concept of “ecological civilization”. Here, we estimated the N footprint and its dynamic changes in the urban and rural areas of Shanghai between 2000 and 2017 using the N-Calculator model, and predicted the N footprint between 2018 and 2027 using the auto regressive integrated moving average (ARIMA) time series model [14]. The ARIMA model is a statistical analysis model that uses time series data to either better understand the data set or to predict future trends; it is used in many fields including finance, environmental science and agriculture [14]. The results are very important for raising residents’ awareness of the need to reduce food waste and reduce reactive N emissions in food production and consumption.

## 2. Materials and Methods

### 2.1. Study Area

Shanghai is located between 120°52′ E–122°12′ E and 30°40′ N–31°53′ N. It has an area of 6340 km^2^, which includes 16 municipal districts, 106 residential districts, 107 towns, and 2 townships [15], and it is one of the most developed regions of China. The number of permanent residents of Shanghai reached 16.1 million in 2000 and 24.2 million in 2017 [16]. In fact, the number of people and the population density has increased annually. In 2017, Shanghai’s GDP was 6.37 times higher than in 2000. In particular, the service industry increased 8.46-fold. Between 2000 and 2017, the per capita disposable annual income of urban and rural residents increased from 11,718 to 57,696 Yuan and from 5565 to 27,825 Yuan, respectively. In this study, a rural resident refers to a permanent resident with a rural household registration, and an urban resident is a permanent resident with an urban household registration in Shanghai. The Engel’s coefficient is the proportion of family income that is spent on food, which decreased from 44.5% to 15.3% for urban residents between 2000 and 2017, while for rural residents it decreased from 44% to 23.6% [15], which indicates a significant improvement in living standards in both cases.

### 2.2. Data Source

The data for per capita food consumption in Shanghai that were used in this study came from the Shanghai Statistical Yearbook [15]. The per capita disposable income, food price, and population size were obtained from the National Statistical Yearbook [16] and the Shanghai Statistical Bulletin on National Economic and Social Development [17]. Other data were obtained documents published between 2000 and 2016. Due to the lack of data on the per capita consumption of urban and rural residents in Shanghai, food N footprint data from 2014 were excluded. In addition, the data for per capita grain consumption by urban residents in 2007–2016 were calculated by dividing Shanghai’s grain purchase expenditure (which was indicated as “average annual consumption expenditure per household in each region” in the National Statistical Yearbook) by the local grain price. The grain price in 2007–2016 was calculated based on the yearly grain retail price indices listed in the Shanghai Statistical Yearbook. Moreover, the N content of different foods and nutritionally balanced diets was collected from different literature [4,10,13].

### 2.3. Calculation Methods

#### 2.3.1. Calculation of the Food N Footprint

**Figure 1:**
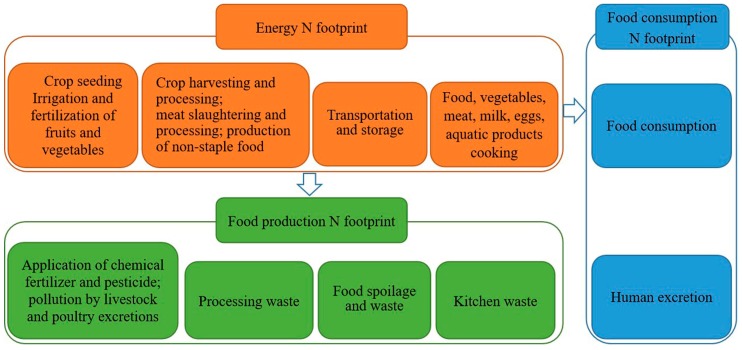
Nitrogen (N) footprint during the food life cycle.

The framework of the N-Calculator model developed by Leach et al. [18] was used to calculate the food N footprint in Shanghai, and mainly considered the production, primary processing, transportation, reprocessing, and consumption phases as described by Xian and Quyang [12] (Figure 1). In this study, livestock and poultry feed was excluded from the food production N footprint due to a lack of data. Similarly, due to a lack of data regarding the consumption of soybean products and the per capita food consumption of local residents when dining-out, they were not included in the energy N footprint calculation. The food N footprint was calculated based on the following equations:

FP_T_ = FPp + FPc + FPe (1)

where FP_T_, FPp, FPc, and FPe indicate the food N, food production N, food consumption N, and the food energy N footprints, respectively.

The FPp corresponds to the total reactive N produced during the food production process [18]. Because N fertilizers are not fully absorbed by crops and N is lost during food processing, part of the reactive N is lost before the final food product is obtained. The term virtual N, indicates the total N introduced into the cycle through the application of crop N fertilizers, the excretions of livestock and poultry, food processing, food spoilage, and kitchen waste generated during cooking. The amount of reactive N released into the environment per kilogram of food corresponds to the virtual N factor [10], which represents the environmental cost of different types of food during different steps [10,11,19]. This virtual N factor can be used to calculate the amount of N lost in the food life cycle before the food consumption phase and it was first used in 2012 to calculate N emissions from food consumption in the United States [11]. In this study it was used to make a preliminary estimation of the food production N footprint before food consumption. The virtual N factors of cereal, vegetables, and fruit used in our study were calculated by Zhang et al. [20], and other virtual N factors including livestock meat, poultry meat, aquatic product, egg and milk were derived from a previous study in the United States [11]. The production N footprint was calculated by multiplying the food consumption N footprint with the food virtual N factor (Table 1) [13,21].

The FPc is equal to the total reactive N produced during the food consumption process [18], and can be obtained by multiplying the per capita food consumption for the food N content (Table 1). Since there is a balance between human N intake and excretion, we assumed that all the food N entering the human body would re-enter the natural environment in the form of manure N [10]. Due to the low total consumption of sugar and bean products in the study area and the lack of relevant data, such products were not included in this study. Hence, we classified the food consumed by the urban and rural residents of Shanghai into eight categories: livestock meat, poultry meat, aquatic products, cereal, vegetables, fruit, eggs, and milk.

The FPe is defined as the energy consumed between the food production and consumption phases. It corresponds to the total reactive N produced during food production, transportation, cooking, and processing [12]. The corresponding N footprint data are numerous and difficult to manage, so in order to simplify their calculation, we compared our food N footprint calculation with that previously done in the United States, and considered 25% of the total N footprint as the energy N footprint [10].

#### 2.3.2. The ARIMA Prediction Model

**Figure 2:**
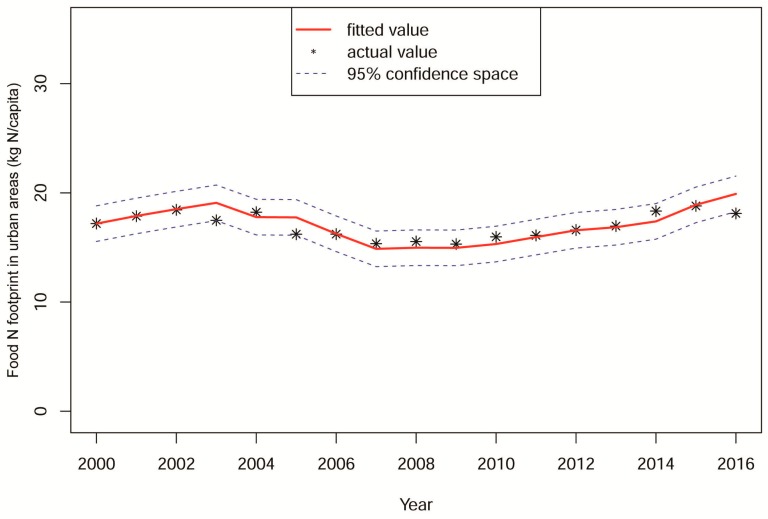
Predicted values fit the graph of the auto regressive integrated moving average (ARIMA) (2, 2, 0) model.

The environmental N load of food consumption in Shanghai in 2018–2027 was predicted by the ARIMA model based on the N footprint time series from 2000 to 2017. The ARIMA model is a short-term prediction model with a high accuracy for random time series. It converts moving time series into stationary time series, and then models them in order to determine the best model [22]. The ARIMA model included the following steps:

(1) Stationary series. Due to the instability of the original time sequence for urban food N footprints per capita from 2000 to2017, a second-order difference was conducted. The results showed that the observed value fluctuated randomly around a mean value of 0, which met the stability requirement.
(2) Determining the values of p, d, and q. The letter p, d, and q indicate the order of the autoregression, difference, and moving average, respectively. Likely p and q values are determined based on the autocorrelation function (ACF) and partial autocorrelation function (PACF) results of the series diagram. The values of p, d, and q were finally determined taking into account that smaller Akaike information criterion (AIC) and Bayesian information criterion (BIC) values correspond to a higher prediction accuracy. Based on the series diagram of the second-order difference, the ACF and PACF diagrams were established. According to the p-order posterior truncation of the PACF (which fell in the confidence space) and the q-order posterior attenuation of the ACF (tending to 0), eight alternative models (i.e., ARIMA (1, 2, 1), ARIMA (1, 2, 2), ARIMA (2, 2, 1), ARIMA (2, 2, 2), ARIMA (0, 2, 2), ARIMA (0, 2, 1), ARIMA (1, 2, 0), and ARIMA (2, 2, 0)) were fitted preliminarily. The prediction model ARIMA (2, 2, 0) was chosen based on the lower values of the associated AIC and BIC and because it had an R^2^ very close to 1.
(3) Model residual test. In order to test the normality of the ARIMA residuals, a white noise test was conducted in the residual series and the p-value was equal to 0.9036, indicating that the residuals sequence was represented by white noise and that the model accuracy was very high. For these reasons, ARIMA (2, 2, 0) could be used effectively for data prediction. A similar prediction method was applied to the rural areas, but using the ARIMA model.
(4) Evaluation of the model prediction. The data predicted by the model were evaluated via comparison with the actual data for the N footprint of Shanghai residents from 2000 to 2017. The fitting value predicted by the ARIMA (2, 2, 0) model was found to be within the 95% confidence space of the predicted N load (Figure 2).

### 2.4. Statistical Analysis

Spearman correlation analysis was applied to the relationships between the per capita food N footprint and food consumption, and between the food N footprint and socioeconomic factors, where *p* < 0.05 and *p* < 0.01 was considered significant and very significant, respectively.

## 3. Results

### 3.1. Estimation of the Food N Footprint of Urban and Rural Residents

The estimated food N footprints of urban and rural residents in Shanghai are listed in Table 2. Except for cereal, the average food N footprint per capita from 2000 to 2017 was higher in urban areas than rural areas. The food N footprint per capita from2000 to 2017 was 1.1, 1.3, 1.3, 1.4, 2.1, 1.2, and 3.6 times higher for urban residents than rural residents for livestock meat, poultry meat, aquatic products, vegetables, fruit, eggs, and milk, respectively. Compared to 2000, the total food N footprint of urban and rural residents in 2017 was higher with 18.5 and 16.9 kg N/capita/yr, respectively. Notably, the non-staple food N footprint per capita of urban residents and the vegetarian food N footprint per capita of rural residents in 2017 were lower compared with that in 2000.

### 3.2. Changes in the Food N Footprint among Urban and Rural Residents

**Figure 3:**
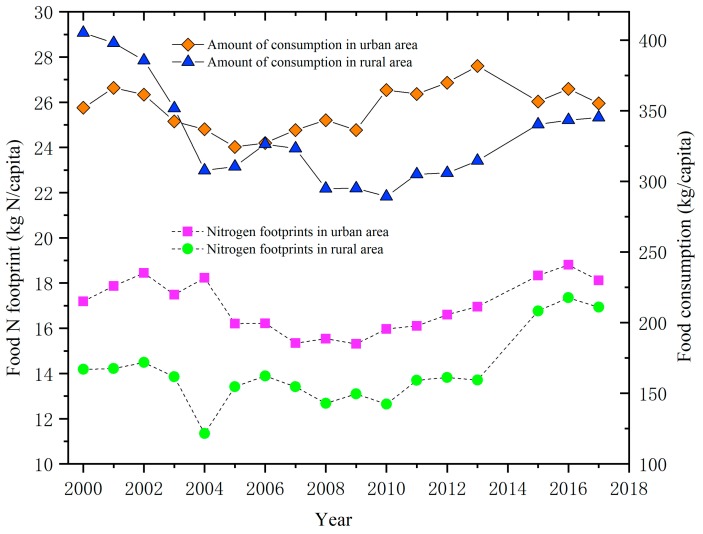
Variation in food consumption and N footprints per capita of Shanghai residents from 2000 to 2017.

The food N footprint of urban residents ranged between 15.3–18.8 kg N/capita/yr from 2000 to 2017, while that of rural residents ranged between 12.6 and 17.4 kg N/capita/yr (Figure 3). The food N footprint reached its lowest value in 2009 in the urban area (15.3 kg N/capita) and in 2004 in the rural area (11.3 kg N/capita). Similar to the food N footprint, food consumption in urban and rural areas first decreased and then increased. The Spearman correlation analysis showed that the per capita food N footprint was significantly correlated to food consumption in both rural (*p* = 0.000, R = 0.816) and urban areas (*p* = 0.024, R = 0.544). However, the per capita food N footprint of urban areas did not decrease with food consumption from 2015 to 2017, but showed an increasing trend (Figure 3).

**Figure 4:**
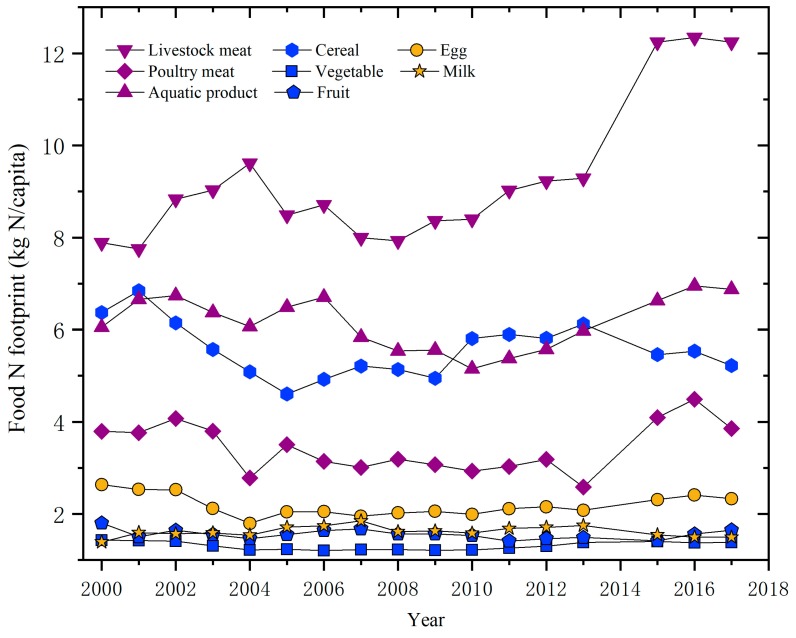
Changes in the nitrogen footprints of different food category in Shanghai from 2000 to 2017.

As shown in Figure 4, the change in the N footprint of livestock meat, aquatic products, cereal, and poultry meat was significant for Shanghai residents from 2000 to 2017. The N footprints of livestock meat, poultry meat, and aquatic products increased significantly from 2015 to 2017, while the cereal N footprint decreased significantly. The change in the vegetable, fruit, egg, and milk N footprints was negligible from 2000 to 2017.

**Figure 5:**
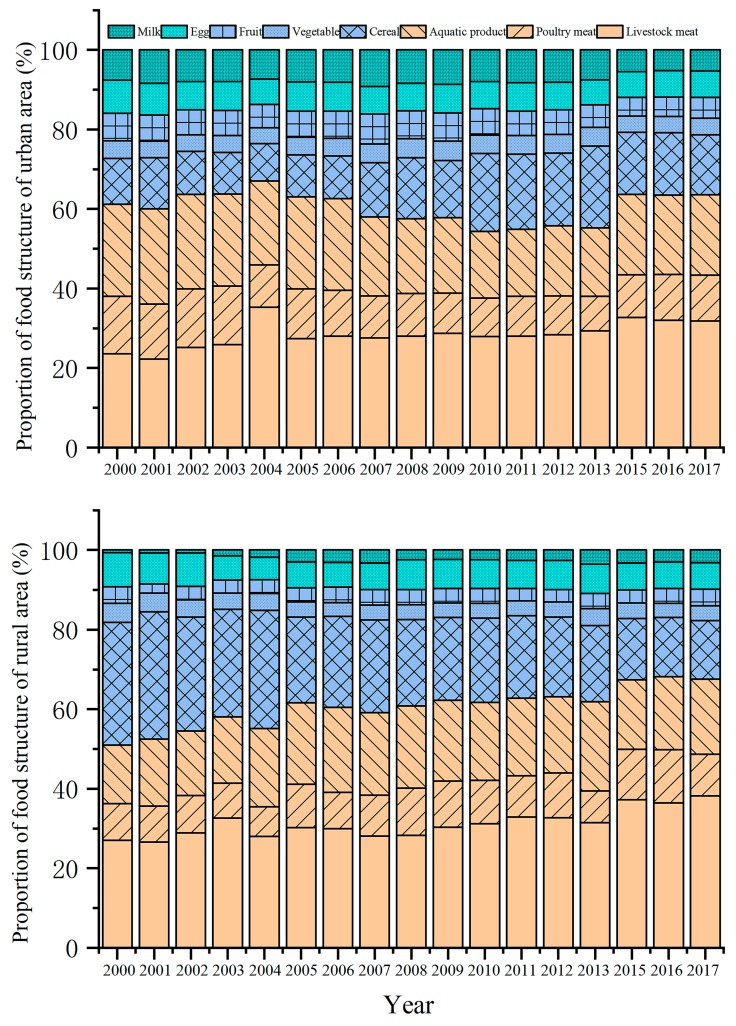
Changes in the proportion of various food N footprints in the urban and rural areas of Shanghai from 2000 to 2017. The food N footprints were analyzed by weight (kg N/capita/yr).

As shown in Figure 5, the proportion of the food N footprint of animal meat (livestock meat, aquatic products, poultry meat), vegetarian food (cereal, vegetable, fruit), and non-staple food (egg and milk) was 54%–67%, 19%–31%, and 11%–17%, respectively, of the total food N footprint in urban areas of Shanghai, while it was 50%–69%, 22%–40%, and 7%–11%, respectively, in the rural areas. The proportion various foods in the structure of urban residents was little changed from 2000 to 2017, while the proportion of vegetarian food in the food structure of rural residents decreased significantly, especially cereal. The proportion of non-staple food was stable, but the proportion of milk increased significantly (Figure 5).

### 3.3. Relationship between the Food N Footprint and Social Economic Factors

Table 3 shows that the total food N footprint of urban residents and rural residents was positively correlated with the per capita disposable income and population, while it was negatively correlated with the Engel’s coefficient and price index. Further analysis showed that the N footprint of aquatic products, cereal, fruit, and milk for urban residents was significantly correlated with the Engel’s coefficient, per capita disposable income, food price index, and population density (Table 3). The N footprint of aquatic products, cereal, and milk for rural residents was significantly correlated with the per capita disposable income and food price index (Table 3).

### 3.4. Prediction of the N Footprint for 2018–2027

The per capita food N footprint in Shanghai residents was predicted for 2018 to 2027 by the ARIMA (2, 2, 0) model (Table 4). The results showed a yearly decrease in the per capita food N footprint in the urban areas of Shanghai between 2018 and 2027. The average value in 2018–2027 was 17.9 kg N/capita/yr, which is lower than that of 2017, while the minimum (17.5 kg N/capita) was predicted for 2027. The per capita food N footprint in the rural areas of Shanghai will gradually increase in 2018–2027 (Table 4). The average predicted value for the next 10-year period was 25.4 kg N/capita/yr, which is higher than that of 2017. Moreover, a maximum value of 27.1 kg N/capita was predicted in 2027.

## 4. Discussion

In our study, the N-Calculator model developed by Leach et al. [10] was used to calculate the food N footprints of urban and rural residents in Shanghai. Due to the difficulty of obtaining data for the consumption of soybean products and the per capita food consumption of local residents when dining-out, these data were not included in the energy N footprint calculations. The virtual N factors for livestock meat, poultry meat, aquatic product, egg and milk were derived from a previous study in the United States, which showed a high food N footprint and a food consumption structure dominated by high-nitrogen types of meat [10,11]. However, due to higher inputs of chemical fertilizer and lower levels of management of fecal and urine wastewater, the N utilization efficiency of crops and animals is lower in China [23,24], which means that the virtual N factor is higher in China than in the United States. Therefore, the food N footprint per capita of urban and rural residents in Shanghai obtained by the N-Calculator model might be smaller than the actual value.

**Figure 6:**
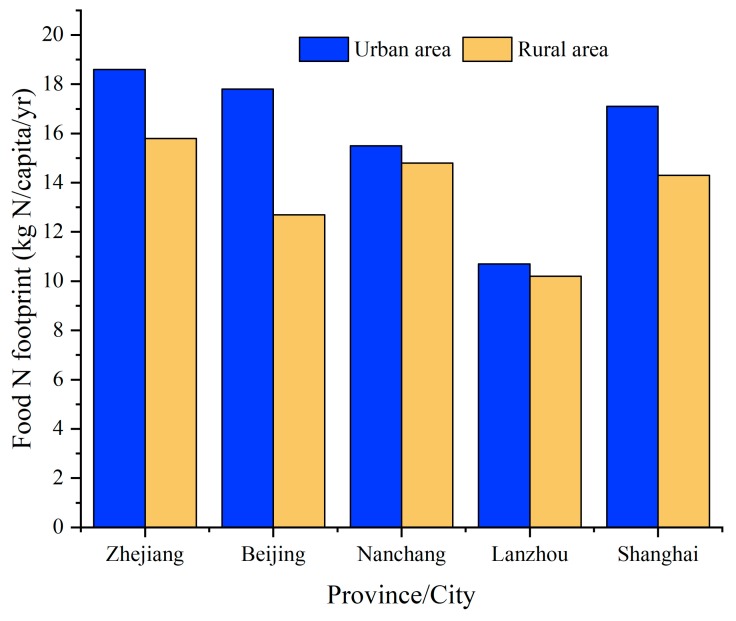
N footprints per capita in urban and rural area in some provinces of China.

To date, the food N footprint of the urban and rural areas of Zhejiang, Beijing, Nanchang, and Lanzhou in China have been reported [12,25,26,27]. As shown in Figure 6, the average food N footprint in urban Shanghai between 2000 and 2017 was 17.1 kg N/capita/yr, which is lower than those calculated for Zhejiang Province between 1980 and 2012 (18.6 kg N/capita/yr) and Beijing City between 2000 and 2014 (17.8 kg N/capita/yr) [12,25], and higher than those obtained for Nanchang City between 1994 and 2014 (15.5 kg N/capita/yr) and Lanzhou City between 2000 and 2014 (10.7 kg N/capita/yr) [26,27]. The average food N footprints per capita of Shanghai rural residents is lower than Zhejiang and Nanchang, but higher than Beijing and Lanzhou (Figure 6). These differences might be due to the close correlation between N footprints and per capita disposable income and population density (Table 3). In addition, it may also be related to the number of years that the food N footprints was calculated in each of the provinces [11]. In 2017, the total food N footprint of rural and urban residents in Shanghai reached 16.9 and 18.5 kg N/capita, respectively, which is close to the average level of 20.0 kg N/capita in Austria [11]. This indicates that consumption patterns in Shanghai are very similar to developed countries and can be characterized by high-N lifestyles [28]. With the development of an ecological civilization in Shanghai and strict control of food consumption, waste classification, and waste treatment by the government, it is expected that the food N footprint per capita of urban residents will gradually decrease as predicted in Table 4.

It has been discovered that the per capita disposable income, Engel’s coefficient, food price index, and average population are closely related with food N consumption [12,29,30]. Per capita disposable income increases with the development of an economy, which to some extent causes a decline in the proportion of vegetarian food and an increase in the proportion of meat that is consumed [31]. Therefore, an increase in the food N footprint can be expected, which was also positively correlated with per capita disposable income in this study (Table 3, Figure 3, Figure 4 and Figure 5). At the same time, economic growth and improvement in people’s living standards reduce the Engel’s coefficient [32], which was negatively correlated with the food N footprint of residents (Table 3). Population density determines the total food consumption [33], which thus showed a positive correlation with the food N footprint (Table 3). In contrast, an increase in the food price index leads to a decrease in purchasing power and consumption [34], which results in a negative correlation with the N footprint of food consumption (Table 3). In addition, compared with 2013, the annual per capita food consumption of urban areas decreased between 2015 and 2017, while the per capita food N footprint of urban areas increased (Figure 3). This could be explained by the increase in the animal meat N footprint that dominates the food N footprint, and the decrease in cereal consumption that dominates food consumption (Figure 5).

Although the estimated food N footprint per capita will decrease in urban areas in the next decade, it will continue to increase significantly in rural areas (Table 4). These results indicate that the food consumption structure of urban residents in Shanghai has changed significantly, that is, they prefer to eat low-nitrogen food, their food consumption is more diverse and their diet structure is more balanced. However, with the development of the rural economy, the income gap between urban and rural residents will be narrowed, and the consumption of high-nitrogen food will gradually increase, resulting in an increasing per capita food N footprint in rural areas (Table 4). Moreover, the predicted total food N footprint of Shanghai residents will continue to increase from 35.8 to 44.6 kg N/capita/yr over the next ten years (Table 4), far exceeding the food N footprint that is typical in developed countries, such as 39 kg N/capita/yr in the United States, 32 kg N/capita/yr in Japan, and 29 kg N/capita/yr in Portugal [11]. In other words, there will be a tendency toward a high-N food consumption mode and structure in Shanghai. There might be some limitations in using the ARIMA model for prediction, such as the selection of time scales, the length of time series, and the correlation of the data selected [24]. Nevertheless, these data should be considered by the government, which should take effective measures to limit the growth of the food N footprint in Shanghai, such as providing financial and policy support, formulating strict standards and implementing standardization, and training professional and technical personnel in order to ensure sustainable services. We propose that Shanghai residents should reduce food waste and participate in the “clean plate action” [35] to reduce the loss of reactive N during food production and consumption. At the same time, the construction of infrastructure for storage and logistics should be improved to reduce the energy N produced during food supply and transportation [11]. Furthermore, from the perspective of balanced diet and the relationship between diet and disease, it is vigorously advocated that we should reduce the proportion of meat in the food structure and increase the proportion of vegetarian and non-staple food.

## 5. Conclusions

Our results show that the food N footprint of urban residents ranged between 15.3–18.8 kg N/capita/yr from 2000 to 2017, while that of rural residents ranged between 12.6–17.4 kg N/capita/yr in Shanghai. These values are comparable with the reported data from other cities in China and are close to other developed countries, and they also reflect the high-N lifestyles of Shanghai. The food N footprint per capita was higher in urban than rural areas of Shanghai, which is mainly caused by the higher proportion of high-N meat and non-staple food consumed in urban compared to rural areas. The total food N footprint of urban and rural residents was positively correlated with the per capita disposable income and population, while it was negatively correlated with the Engel’s coefficient and price index. It was estimated that the per capita food N footprint will gradually decrease between 2018 and 2027 in urban areas of Shanghai, but it will generally increase in rural areas. As the change in the food N footprint was consistent with that of food consumption in urban and rural areas, we propose that residents should reduce the proportion of meat they consume, reduce food waste, and participate in the “clean plate action” to reduce the loss of reactive N during food production and consumption.

## Figures and Tables

**Table 1:** N content and the virtual N factors in food.

Item	N Content (g kg^−1^)	Virtual N Factor
Livestock meat	29.2	4.7
Poultry meat	29.9	3.4
Aquatic product	28.8	3.0
Cereal	14.4	1.0
Vegetable	1.8	2.3
Fruit	1.6	7.1
Egg	20.5	3.4
Milk	5.3	5.7

**Table 2:** The food N footprints of residents in Shanghai in 2000, 2017, and the average value from 2000 to 2017.

Food Category	Urban Food N Footprint (kg N/capita)			Rural Food N Footprint (kg N/capita)		
	2000	2017	Average Value 2000–2017	2000	2017	Average Value 2000–2017
**Animal food**	10.6	11.6	10.3	7.2	11.5	8.6
Livestock meat	4.1	5.8	4.8	3.8	6.5	4.4
Poultry meat	2.5	2.1	2.0	1.3	1.8	1.5
Aquatic Product	4.0	3.7	3.5	2.1	3.2	2.7
**Vegetarian food**	4.0	4.4	4.3	5.7	3.8	4.3
Cereal	2.0	2.7	2.4	4.4	2.5	3.2
Vegetable	0.8	0.8	0.8	0.7	0.6	0.6
Fruit	1.2	0.9	1.1	0.6	0.7	0.5
**Non-staple food**	2.7	2.5	2.5	1.3	1.7	1.4
Egg	1.4	1.2	1.2	1.2	1.1	1.0
Milk	1.3	1.3	1.3	0.1	0.5	0.4
Total	17.3	18.5	17.1	14.2	16.9	14.3

**Table 3:** Correlation coefficients between food N footprints per capita and various economic factors in Shanghai from 2000 to 2017.

Area	Item	Engel’s Coefficient	Per Capita Disposable Income	Food Price Index	Population Density
Urban	Livestock meat	−0.495 *	0.567 *	−0.322	0.472
	Poultry meat	0.459	−0.483 *	−0.514 *	−0.577 *
	Aquatic product	0.568 *	−0.637 **	−0.577 *	−0.700 *
	Cereal	−0.505 *	0.721 **	0.512 *	0.772 **
	Vegetable	0.028	0.289	0.047	0.399
	Fruit	0.768 **	−0.892 **	−0.610 **	−0.816 **
	Egg	0.420	−0.432	−0.620 *	−0.498 *
	Milk	0.713 **	−0.679 **	−0.535 *	−0.689 **
	Total	−0.018	0.022	−0.136	0.145
Rural	Livestock meat	−0.471	−0.642 **	0.107	0.298
	Poultry meat	−0.304	0.664 **	0.195	−0.096
	Aquatic product	−0.311	0.787 **	0.587 **	−0.197
	Cereal	0.368	−0.953 **	−0.666 **	0.094
	Vegetable	−0.116	−0.113	−0.306	0.417
	Fruit	0.328	0.385	0.216	−0.303
	Egg	0.002	0.206	−0.083	0.207
	Milk	0.388	0.838 **	0.642 **	−0.168
	Total	−0.348	0.181	−0.161	0.387

** means that the significance level is less than 1%; * means that the significance level is less than 5%.

**Table 4:** Predicted value of food N footprints in Shanghai from 2018 to 2027.

Year	Predicted Value (kg N/capita)		Confidence Space of 95%			
			Upper Limit (kg N/capita)		Lower Limit (kg N/capita)	
	Urban	Rural	Urban	Rural	Urban	Rural
2018	18.5	17.3	20.8	17.6	16.1	17.1
2019	18.1	19.8	21.7	20.6	14.5	19.0
2020	18.2	20.1	24.0	21.7	12.4	18.5
2021	18.0	22.2	25.9	24.6	10.1	19.9
2022	18.0	22.5	28.4	25.6	7.1	19.3
2023	17.8	23.3	31.0	27.2	4.7	19.3
2024	17.8	25.3	33.8	30.2	1.7	20.4
2025	17.7	25.6	36.8	31.6	−1.5	19.6
2026	17.6	26.2	40.0	32.3	−4.8	18.1
2027	17.5	27.1	43.3	35.5	−8.3	18.8
